# Synthesis and Performance of Aromatic Polyamide Ionenes as Gas Separation Membranes

**DOI:** 10.3390/membranes10030051

**Published:** 2020-03-22

**Authors:** Kathryn E. O’Harra, Irshad Kammakakam, Danielle M. Noll, Erika M. Turflinger, Grayson P. Dennis, Enrique M. Jackson, Jason E. Bara

**Affiliations:** 1 Department of Chemical & Biological Engineering, University of Alabama, Tuscaloosa, AL 35487-0203, USA; keoharra@crimson.ua.edu (K.E.O.); ikammakakam@eng.ua.edu (I.K.); dmnoll@crimson.ua.edu (D.M.N.); emturflinger@crimson.ua.edu (E.M.T.); gpdennis@crimson.ua.edu (G.P.D.); 2 NASA Marshall Space Flight Center, Huntsville, AL 35812, USA; enrique.m.jackson@nasa.gov

**Keywords:** aromatic polyamides, ionic liquids, ionenes, membranes, gas separations

## Abstract

Here, we report the synthesis and thermophysical properties of seven primarily aromatic, imidazolium-based polyamide ionenes. The effects of varied *para*-, *meta*-, and *ortho*-connectivity, and spacing of ionic and amide functional groups, on structural and thermophysical properties were analyzed. Suitable, robust derivatives were cast into thin films, neat, or with stoichiometric equivalents of the ionic liquid (IL) 1-benzy-3-methylimidazolium bistriflimide ([Bnmim][Tf_2_N]), and the gas transport properties of these membranes were measured. Pure gas permeabilities and permselectivities for N_2_, CH_4_, and CO_2_ are reported. Consistent *para*-connectivity in the backbone was shown to yield the highest CO_2_ permeability and suitability for casting as a very thin, flexible film. Derivatives containing terephthalamide segments exhibited the highest CO_2_/CH_4_ and CO_2_/N_2_ selectivities, yet CO_2_ permeability decreased with further deviation from consistent *para*-linkages.

## 1. Introduction

Gas separation membranes consisting of polymeric materials have drawn significant research attention, as the technology and materials are scalable and performance competes with more energy-intensive conventional techniques including pressure swing adsorption, cryogenic distillation, and gas–liquid absorption [1,2,3,4]. Materials considered to possess state-of-the-art gas separation performance feature certain functional and structural moieties, which may impart higher free volume distribution or promote flexibility and thermal stability. Polyimides [5,6,7,8,9,10], polymers of intrinsic microporosity (PIMs) [11,12], thermally-rearranged polymers (TR) [13,14,15,16], polyamides [17,18,19], and ion-containing polymers [20,21] are classes of polymeric materials which have been prevalent in this research area. Polyamides are another desirable material class as they feature H-bonding, which often produce self-assembled or well-ordered materials due to chain alignment [22,23,24]. 

Polyamide membranes and related composites are versatile, and perform well at various separation scales. Amide functionality in polymers has been shown to improve the mechanical strength, chemical and thermophysical stability due to the H-bonding and the induced ordering of polymer chains. Certain polyamides exhibit interesting surface behaviors, rendering a platform amenable to post-polymerization modifications. These surface interactions have been studied to show suitability for grafting or deposition upon other frameworks [25,26,27,28,29], or harnessed as antifouling and antimicrobial coatings [30,31,32]. Polyamide films have been explored for nanofiltration, reverse/forward osmosis, desalination [33,34], gas separations [17,35,36,37,38,39], and wastewater treatment [40]. The structuring and chain packing of the polyamide matrix is also impacted by the preparation technique, as films have been processed by solution casting [17,35], layer-by-layer fabrication [41,42], 3D-printing [43,44], and interfacial polymerization [34,45,46] methods. Tailoring polymer structure and morphology via rational molecular design is important to advancing progress in membrane-based separations, as well as improving mechanical and thermal properties.

Recently, we have synthesized polyimide ionene (PI-ionene) and polyamide ionene (PA-ionene) materials for gas sepration membranes, with the combination of functional groups and ionic components yielding flexible yet robust thin films via solvent-casting or melt-pressing. These ionenes incorporate imidazolium cations within the main backbone paired with “free” (i.e., untethered) fluorinated anions, spacing functional groups or high free volume elements [6,8,47]. Ionenes are an form of ionic polymers that is typically synthesized via condensation reactions [48]. The chain entanglement and organization in these PA-ionenes are dictated by ionic, H-bonding, π-cation and π–π stacking intermolecular interactions [49]. The contributions of these noncovalent interactions influence self-assembly and structuring of the matrix, which can be further tuned by adding “free” ionic liquids (ILs) to the PA-ionenes. 

This work presents the synthesis of five new highly aromatic amide-linked bis(imidazole) monomers, which were subsequently polymerized via Menshutkin reactions yielding a set of imidazolium PA-ionene isomers with systematically varied aromatic connectivity/regiochemistry. Select derivatives were impregnated with IL, which acts as a non-volatile plasticizer, in order to probe the impact of these ionic interactions on nanostructuring and chain alignment as well as membrane fabrication. The effects of alternating connectivity between functional and charged features on chain packing and structuring are discussed, and the performance and gas transport properties of several solvent-cast films are analyzed. 

## 2. Materials and Methods 

### 2.1. Materials

Terephthaloyl chloride (TC, >99%), α, α’-Dichloro-p-xylene (pDCX, >98%), α, α’-Dichloro-m-xylene (mDCX, >96%), and α, α’-Dichloro-o-xylene (oDCX, >97%) were purchased from TCI (Portland, OR, USA). Isophthaloyl chloride (IC, 98%) and potassium carbonate (K_2_CO_3,_ 99% anhydrous) were purchased from BeanTown Chemical (Hudson, NH, USA). Lithium bistriflimide (LiTf_2_N) was purchased from 3M (Minneapolis, MN, USA). Acetonitrile (CH_3_CN, ACS Grade) and N-methylpyrrolidone (NMP, ACS Grade) were purchased from VWR (Atlanta, GA, USA). 

### 2.2. Characterization

^1^H-NMR and ^13^C-NMR spectra were obtained using 360 MHz or 500 MHz Bruker Avance instruments (Madison, WI, USA). The thermal stabilities of these PA-ionenes were evaluated by thermogravimetric analysis (TGA) at a heating rate of 10 °C·min^−1^ under a N_2_ atmosphere (Seiko TG/DTA 7300). The glass transition temperature (*T*_g_) of each PA-ionene was observed by DSC (TA Instruments, DSC Q200) (New Castle, DE, USA) from 30–300 °C with a scan rate of 10 °C·min^−1^ under N_2_. The wide-angle X-ray diffraction (WAXD) patterns of the materials were measured using a Bruker D8 Discover diffractometer by employing a scanning rate of 4° min^−1^ in a 2θ range from 5 to 70° with a Co Kα_1_ X-ray source (λ = 0.17886 nm). The *d*-spacing values were calculated using Bragg’s law (d = λ/2sin θ) and Diffrac-EVA software (Bruker). Number average molecular weight (M_N_) values of the PA-ionenes were determined via MALDI-TOF MS (Bruker Ultraflex).

### 2.3. Synthesis of Bis-Imidazole Monomers Containing Amide Functionality

#### 2.3.1. Synthesis of 4-(1H-imidazol-1-yl)aniline “I4A”, 3-(1H-imidazol-1-yl)aniline “I3A”, and 2-(1H-imidazol-1-yl)aniline “I2A”

The synthesis of 4-(1H-imidazol-1-yl)aniline (I4A) starting from imidazole and 4-fluoronitrobenzene followed by reduction in EtOH with H_2_ over Pd/C was reported in our previous work [47]. ^1^H NMR (500 MHz, DMSO-*d*_6_) δ 7.95 (s, 1H), 7.48 (s, 1H), 7.22 (d, 2H), 7.01 (s, 1H), 6.64 (d, J = 8.8, 2H), 5.26 (s, 2H). The synthesis of 3-(1H-imidazol-1-yl)aniline “I3A” was synthesized via a similar procedure and was outlined in our previous work [6]. ^1^H NMR (360 MHz, DMSO-*d*_6_) δ 8.08 (s, 1H), 7.57 (t, *J* = 1.3 Hz, 1H), 7.13 (t, *J* = 1.3 Hz, 1H), 7.08 (s, 2H), 6.73 (t, 1H), 6.72–6.67 (m, 1H), 6.60–6.53 (m, 1H), 5.41 (s, 2H). The synthesis of 2-(1H-imidazol-1-yl)aniline “I2A” was synthesized via a similar procedure and was outlined in our previous work [47]. ^1^H NMR (360 MHz, DMSO-*d*_6_) δ 7.74 (t, J = 1.1 Hz, 1H), 7.30 (t, J = 1.3 Hz, 1H), 7.14 (ddd, J = 8.1, 7.3, 1.6 Hz, 1H), 7.10 (t, J = 1.1 Hz, 1H), 7.03 (dd, J = 7.8, 1.6 Hz, 1H), 6.86 (dd, J = 8.1, 1.4 Hz, 1H), 6.64 (td, J = 7.5, 1.4 Hz, 1H), 5.06 (s, 2H).

#### 2.3.2. Synthesis of Bis-Imidazole Amide Monomers

**Scheme 1:**
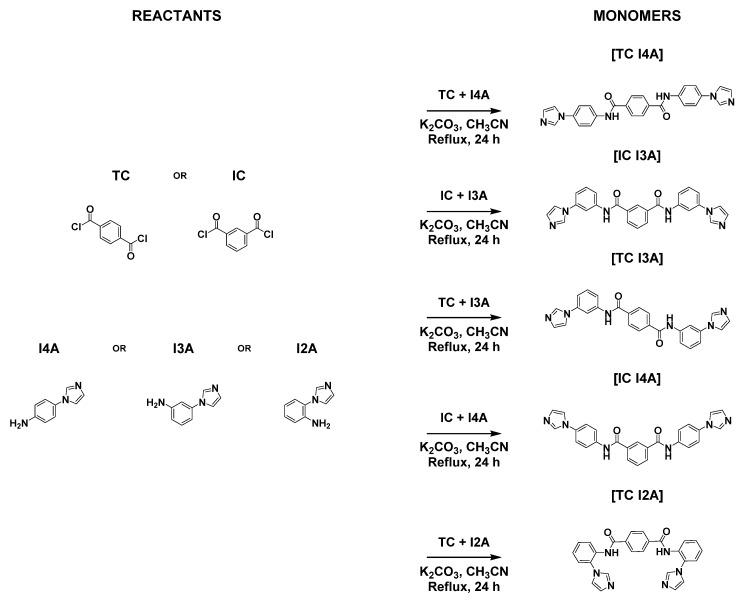
Synthesis of bis-imidazole monomers containing amide groups formed via condensation.

Five bis-imidazole monomers were synthesized, via condensation of 2 eq. of I4A, I3A, or I2A with 1 eq. TC or IC to form the amide linkage, according to Scheme 1. 

TC (6.96 g, 34.3 mmol), I4A (12.0 g, 75.4 mmol), and K_2_CO_3_ (20.8 g, 150.7 mmol) were added to a 500 mL round-bottom-flask. CH_3_CN (150 mL) was added, and the flask was equipped with a stir bar and reflux condenser. The reaction was heated to 85 °C for 24 h. The mixture was allowed to cool to RT, and then was poured into 350 mL of deionized (DI) water. The precipitate was filtered and washed with 2 × 50 mL of deionized water, then dried in a vacuum oven at 100 °C for 24 h to yield the product “TC I4A” as a white powder (10.2 g, 67%). ^1^H NMR (360 MHz, DMSO-*d*_6_) δ 10.60 (s, 2H), 8.26 (s, 2H), 8.16 (s, 4H), 7.97 (d, *J* = 8.8 Hz, 4H), 7.76 (s, 2H), 7.69 (d, *J* = 8.8 Hz, 4H), 7.13 (s, 2H).

IC (4.93 g, 24.3 mmol), I3A (8.5 g, 53.4 mmol), and K_2_CO_3_ (14.8 g, 106.8 mmol) were reacted in 120 mL of CH_3_CN via a similar procedure to yield the product “IC I3A” as a white powder (10.4 g, 95%). ^1^H NMR (500 MHz, DMSO-*d*_6_) δ 10.69 (s, 2H), 8.60 (s, 1H), 8.20 (d, *J* = 6.0 Hz, 4H), 8.08 (s, 2H), 7.81 (d, *J* = 8.1 Hz, 2H), 7.75 (t, *J* = 7.7 Hz, 1H), 7.68 (s, 2H), 7.53 (t, *J* = 8.1 Hz, 2H), 7.43–7.38 (m, 2H), 7.15 (s, 2H).

TC (4.93 g, 24.3 mmol), I3A (8.5 g, 53.4 mmol), and K_2_CO_3_ (14.8 g, 106.8 mmol) were reacted in 120 mL of CH_3_CN via a similar procedure to yield the product “TC I3A” as a white powder (10.3 g, 94%). ^1^H NMR (500 MHz, DMSO-*d*_6_) δ 10.63 (s, 2H), 8.20 (s, 2H), 8.15 (br, 4H), 8.08 (s, 2H), 7.80–7.77 (m, 2H), 7.69 (s, 2H), 7.57–7.53 (m, 2H), 7.43–7.39 (m, 2H), 7.15 (s, 2H).

IC (4.93 g, 24.3 mmol), I4A (8.5 g, 53.4 mmol), and K_2_CO_3_ (14.8 g, 106.8 mmol) were reacted in 120 mL of CH_3_CN via a similar procedure to yield the product “IC I4A” as a white powder (10.7 g, 97%). ^1^H NMR (500 MHz, DMSO-*d*_6_) δ 10.62 (s, 2H), 8.59 (s, 2H), 8.24 (s, 2H), 8.19 (dd, *J* = 7.7, 1.7 Hz, 2H), 7.96 (d, *J* = 8.9 Hz, 4H), 7.74 (m, 4H), 7.67 (m, 4H), 7.11 (s, 2H).

TC (6.96 g, 34.3 mmol), I2A (12.0 g, 75.4 mmol), and K_2_CO_3_ (20.8 g, 150.7 mmol) were reacted in 150 mL of CH_3_CN via a similar procedure to yield the product “TC I2A” as a brown powder (2.07 g, 42%). ^1^H NMR (360 MHz, DMSO-*d*_6_) δ 13.13 (br, 2H) 8.93–8.84 (m, 2H), 8.46 (t, *J* = 7.7 Hz, 2H), 8.17–7.95 (m, 6H), 7.85–7.75 (m, 6H), 7.75–7.69 (m, 2H).

### 2.4. Synthesis of PA-Ionene Isomers

**Figure 1:**
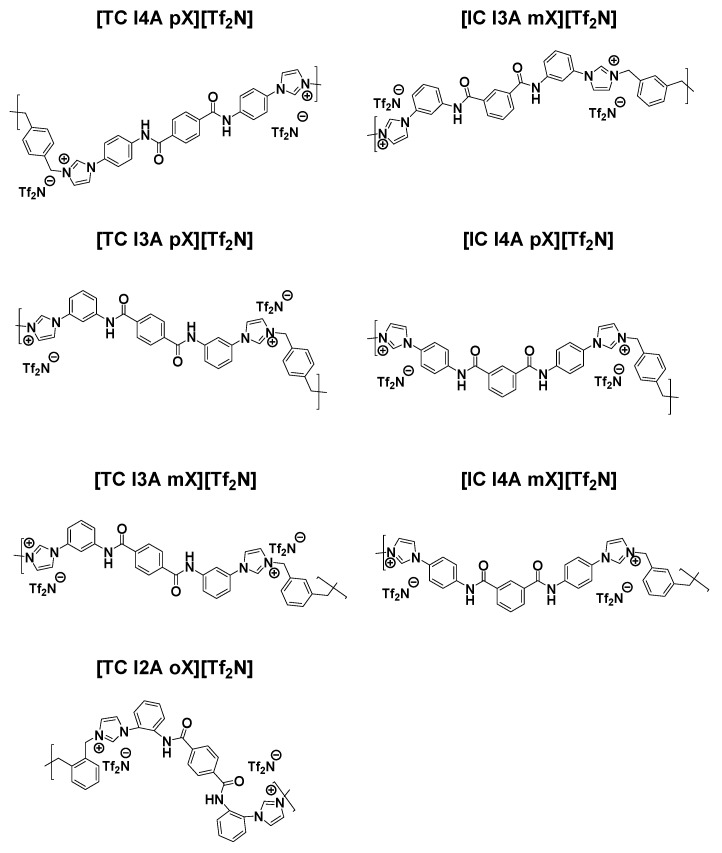
Seven PA-ionenes synthesized and studied in this work.

Seven PA-ionenes (Figure 1) were formed via the Menshutkin reaction, from bis-imidazole amide monomers with pDCX, mDCX, or oDCX.

The PA-ionenes were formed from equimolar amounts (1:1) of the respective bis-imidazole monomers and DCX derivatives. The monomers were added with 200 mL of NMP and a stir bar to a 500 mL round-bottom heavy-walled pressure vessel (Ace Glass, Vineland, NJ, USA) and sealed with PTFE screw caps. The reaction was heated at 150 °C for 24 h. Following polymerization, the reaction mixture was poured into a flask containing 500 mL DI water containing 2.5 eq. of LiTf_2_N under vigorous mechanical stirring to promote ion exchange from the [Cl^‒^] to the [Tf_2_N^‒^] form. The ionenes precipitate as a coarse powder which was collected by vacuum filtration, washed with 2 × 100 mL DI water, then collected to dry in a vacuum oven for 24–48 h at 100 °C. For brevity, the gram/molar amounts of each monomer and the respective yields for each PA-ionene are outlined in Table 1. 

Many peaks in the ^1^H-NMR spectra for the PA-ionene are broad, due to the polymeric character. [TC I4A pX][Tf_2_N]: ^1^H NMR (500 MHz, DMSO-*d*_6_) δ 10.75 (s, 2H), 9.98 (s, 2H), 8.33 (br, 2H), 8.17 (br, 4H), 8.07 (br, 4H), 8.01 (br, 2H), 7.82 (br, 4H), 7.61 (s, 4H), 5.53 (br, 4H). [IC I3A mX][Tf_2_N]: ^1^H NMR (500 MHz, DMSO-*d*_6_) δ 10.89 (s, 2H), 9.98 (s, 2H), 8.43 (br, 2H), 8.34 (br, 2H), 8.27-8.09 (br, 4H), 8.02 (br, 2H), 7.89–7.73 (br, 4H), 7.69 (s, 2H), 7.54 (br, 4H), 5.56 (s, 4H). [TC I3A pX][Tf_2_N]: ^1^H NMR (360 MHz, DMSO-*d*_6_) δ 10.87 (s, 2H), 10.03 (s, 2H), 8.47 (br, 2H), 8.34 (br, 2H), 8.19 (br, 4H), 8.05 (br, 2H), 7.82 (br, 2H), 7.71 (br, 2H), 7.64 (br, 4H), 7.56 (d, *J* = 7.1 Hz, 2H), 5.56 (s, 4H). [TC I3A mX][Tf_2_N]: ^1^H NMR (360 MHz, DMSO-*d*_6_) δ 10.90 (s, 2H), 10.04 (s, 2H), 8.49 (br, 2H), 8.37 (br, 2H), 8.19 (d, *J* = 4.4 Hz, 4H), 8.06 (br, 2H), 7.85 (d, *J* = 7.9 Hz, 2H), 7.71 (t, *J* = 7.7 Hz, 2H), 7.56 (br, 6H), 5.58 (s, 4H). [IC I4A pX][Tf_2_N]: ^1^H NMR (360 MHz, DMSO-*d*_6_) δ 10.85 (s, 2H), 10.00 (s, 2H), 8.64 (br, 2H), 8.34 (br, 2H), 8.25 (br, 2H), 8.10 (br, 4H), 8.02 (br, 2H), 7.88–7.78 (m, 4H), 7.62 (s, 4H), 5.54 (s, 4H). [IC I4A mX][Tf_2_N]: ^1^H NMR (500 MHz, DMSO-*d*_6_) δ 10.80 (s, 2H), 9.96 (s, 2H), 8.60 (br, 2H), 8.36 (br, 2H), 8.23 (br, 2H), 8.09 (br, 4H), 8.02 (br, 2H), 7.89 (d, *J* = 9.5 Hz, 1H), 7.83 (d, *J* = 7.5 Hz, 4H), 7.78 (s, 1H), 7.53 (br, 4H), 5.55 (s, 4H). [TC I2A oX][Tf_2_N]: ^1^H NMR (360 MHz, DMSO-*d*_6_) δ 10.76 (d, *J* = 15.1 Hz, 2H), 9.90 (m, 2H), 8.13 (br, 2H), 8.04–7.90 (m, 4H), 7.88–7.77 (m, 4H), 7.73 (br, 2H), 7.64 (br, 4H), 7.37–7.09 (m, 4H), 5.68 (s, 4H).

### 2.5. Gas Separation Measurements 

Ideal (i.e., single gas) permeation measurements of membranes formed from select PA-ionenes were performed using a high-vacuum time lag apparatus based on the constant-volume/variable-pressure method, as described in our previous works [6,8,17,47]. To avoid membrane fracture at the edges due to the pressure of the O-ring within the permeation unit, the membranes were masked on both sides using an adhesive aluminum tape in order to confine gas permeation through a fixed membrane area of ½” diameter (A = 0.196 in^2^). These masked membranes were prepared from folded aluminum tape which was punched using a 47 mm punch to create two discs. The edges of the two circles were aligned and a ½” hole was punched in the center, and the membrane was sealed between adhesive layers. The aluminum ‘tabbing’ method has been shown to have no effect on the permeability versus using a full membrane [17,50]. All permeation measurements were conducted at 20 °C and the feed pressure was ~3 atm (~45 psia) against initial downstream vacuum (<0.01 psia). Before each measurement, both the feed and the permeate sides were thoroughly evacuated to remove any residual gases. Pressures and temperatures of both the upstream and downstream volumes were monitored and recorded using the most recent version of LabVIEW software (National Instruments). Using these data, the permeability coefficient of each membrane was determined from the linear slope of the plot of downstream pressure rise relative to time (d*p*/d*t*) according to the following equation:

(1) $$P=\frac{273}{76}\times \frac{Vl}{ATp_{0}}\times \frac{dp}{dt}$$

where *P* is the permeability expressed in barrer (1 barrer = 10^−10^ cm^3^ (STP) cm cm^−2^ s^−1^ cmHg^−1^); *V* (cm^3^) is the downstream volume; *l* (cm) is the thickness of the membrane; *A* (cm^2^) is the effective gas permeation area of the membrane; *T* (K) is the measurement temperature; *p*_0_ (Torr) is the pressure of the feed gas at the upstream and d*p*/d*t* is the rate of the steady-state pressure rise. The ideal permselectivity *(α_A_*_/*B*_*)* for a pair of gases (*A* and *B*) was calculated from the ratio of the individual gas permeability coefficients as, *P_A_*/P_B_.

## 3. Results & Discussion

### 3.1. Characterization of Materials

**Figure 2:**
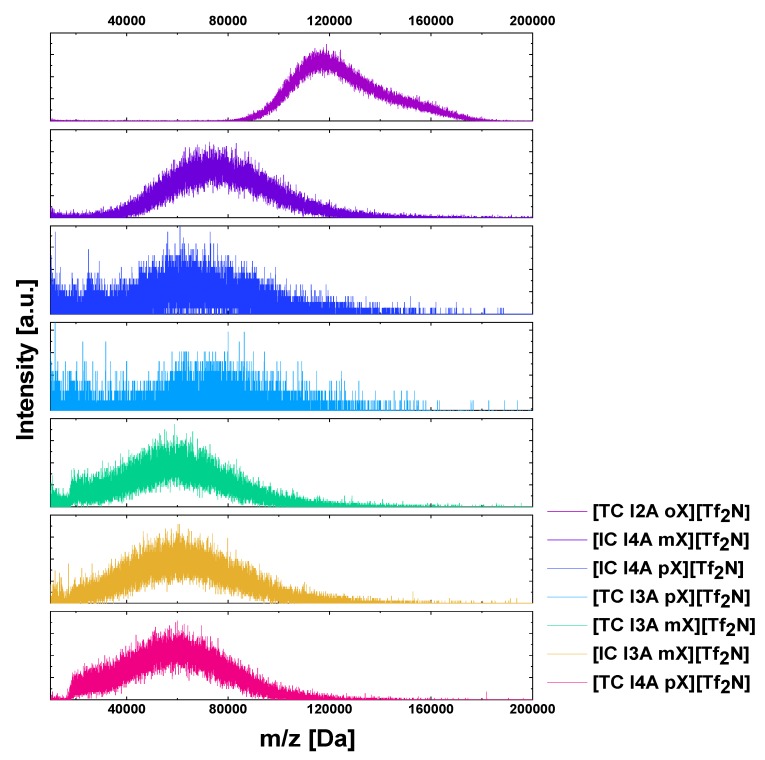
MALDI-TOF MS data for all neat PA-ionene derivatives.

All PA-ionenes were thoroughly characterized, to quantify polymer properties prior to membrane formation and testing. This allows for correlation of repeat unit structure and backbone connectivity with polymer properties. As demonstrated in the supporting information, the purity and structures of all the monomers and polymers were first confirmed by ^1^H-NMR (Figures S1–S12). Density data were collected for each polymer and reported in Table 2. The density measurements were performed using a Mettler Toledo Density Kit (Product MS-DNY-54), paired with a Mettler Toledo analytical balance. This kit allows for solid state density determinations, which is based on Archimedes’ principle. Bulk samples were prepared (dried thoroughly) and tested in triplicate. The samples were weighed at room temperature in air and then in a liquid of known density, in this case, heptane. It should be noted that these ionenes are completely insoluble in heptane nor do they show any swelling/solvent interactions. The measurement was performed using the buoyancy method, and calculations were based on the following equation,

(2) $$\rho_{polymer}=\frac{W_{0}}{W_{0}-W_{1}}\times \rho_{liquid}$$

where *W*_0_ and *W*_1_ are the membrane weights in air and heptane, respectively. The uncertainty for these measurements ±0.01 g [51]. The molecular weight of these ionenes was determined using MALDI-TOF MS experiments, as summarized in Table 2 and included in Figure 2. The thermal transitions and degradation of these PA-ionenes was also investigated using DSC and TGA techniques, and obtained *T*_g_ values are included in Table 2 (Figures S14–S16).

#### 3.1.1. Structural Characterizations

M_N_ values were determined via MALDI-TOF MS (Figure 2) and range from 60–118 kDa, confirming that these combinations of monomers are amenable to the formation of high molecular weight polymers. Given these M_N_ values and utilizing the repeat unit molecular weight (1112.91 g/mol, including “free” anions), number average degree of polymerization ($\bar{X_{N}}$) values of 54–104 repeat units were calculated. These X_N_ values are consistent with those calculated by ^1^H-NMR end group analysis using the ratio of residual dichloro-xylene peaks relative to the polymerized xylyl linkage, which results in a shift of the benzylic CH_2_ protons from ~4.3–4.6 ppm to ~5.5–5.6 ppm. These shifts are pronounced and clear, although integration and comparison of the shifting proton peaks for the imidazole to imidazolium transformation could also be used. From these integration-based ratios, conversion (ρ) and thus $\bar{X_{N}}$ were derived using Carothers’ equation, with calculted X_N_ values between 50–100.

**Figure 3:**
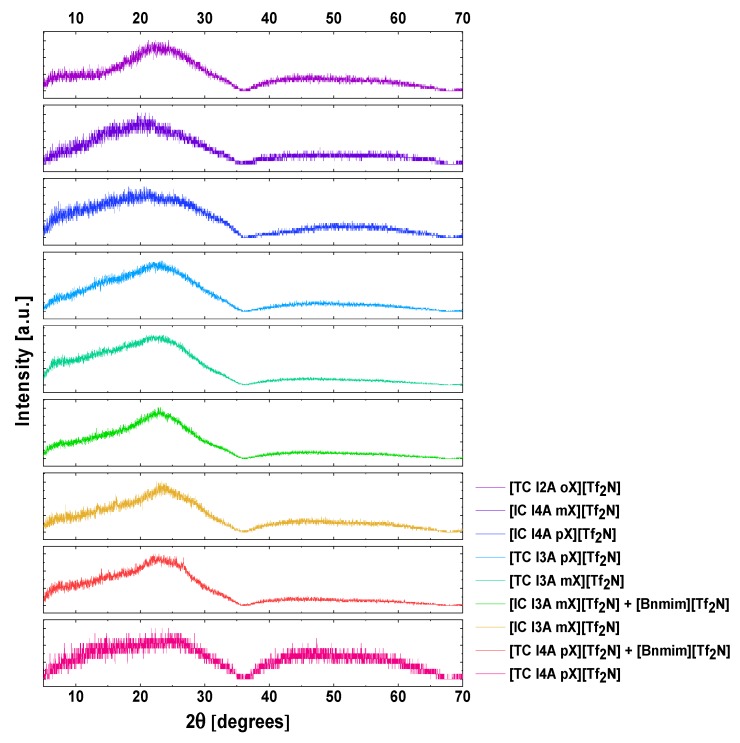
XRD profiles for all PA-ionene derivatives.

The structure was also probed using XRD, as the corresponding *d*-spacing values from these profiles have been associated with estimation of interchain spacing in polymers, which is influential with respect to film formation and gas permeability. Two broad halos are observed for all derivatives in the XRD profiles obtained in the 5°–70° 2θ spectrum, as shown in Figure 3. Typically, polymers do not show defined sharp peaks indicative of significant crystallinity in the bulk material, but instead, show a distribution in the form of an amorphous peak. The center of the distribution, or “peak”, of the main halo is centered between 2θ = 20.2°–25.4°, determined using Diffrac-EVA software. Using Bragg’s law, the corresponding *d*-spacing were calculated in the range of 4.07–5.09 Å. These diffraction profiles support that the distribution of *d*-spacings are similar for this set of PA-ionenes, and reasonable in comparison to other polyamides in literature [52]. It has been shown that *d*-spacing values are representative of interchain spacing and can be used as an approximation for the distance between polymer chains [53]. The intersegmental distances are very closely correlated with derivatives in which the same monomer is used, showing that the connectivity of the xylyl linkage is not as influential. The addition of IL shows a consistent increase in *d*-spacing and sharpening of the main halo peak, which suggests that the effects of IL may lead to greater regularity in the self-assembly and structuring of the matrix. Derivatives **3–6** have mixed connectivity and exhibit the highest *d*-spacing values, which is supported by increased gas permeability when compared to more linear derivatives with consistent connectivity such as **1**. No apparent trend was observed when analyzing the density values, though all fell within a consistent range. However, the rigidity and inherent strain of the *ortho*-connectivity demonstrated in polymer **7** must greatly influence the regularity of chain packing, indicated by the highest density and narrowest XRD halo.

#### 3.1.2. Thermal Characterizations

The thermal behaviors of each material were analyzed using DSC to probe thermal transitions and TGA to determine stability/degradation. Full DSC cycles were run multiple times with polymers which had been dried for 72 h at 100 °C. Precise *T*_g_ and *T*_m_ values for these ionenes were difficult to extract due to relatively broadened transitions possibly due to the complex combination of intermolecular interactions. As indicated in Table 2, *T*_g_ values for these ionenes range from 54–136 °C. These *T*_g_ values were identified through analysis of multiple scans, and correlation of the endotherm on the ramp from 30–300 °C to the corresponding exotherm on the ramp from 300–30 °C (Figures S14 and S15). While the DSC scans were only run up to 300 °C to avoid approaching degradation, it can be seen that each ionene exhibits another broad transition, suggesting *T*_m_ values in the range of 200–320 °C. From TGA analysis, these PA-ionenes are stable at elevated temperatures, though certain cases indicate insufficient drying of these samples. Though some mass loss under 200 °C may have occurred due to the removal of residual trapped solvents, which would imply an underestimation of *T*_d_, these polymers exhibit *T*_d, 10%_ values in the range of 290–360 °C (Figure S16). Generally, the more stable derivatives have consistent connectivity along the backbone (i.e., TC I4A pX, or *p-p-p*), rather than a mix of *para*-*, meta*-, and *ortho*-connectivity (i.e., TC I3A pX, or *p-m-p*). 

### 3.2. Membrane Fabrication

**Figure 4:**
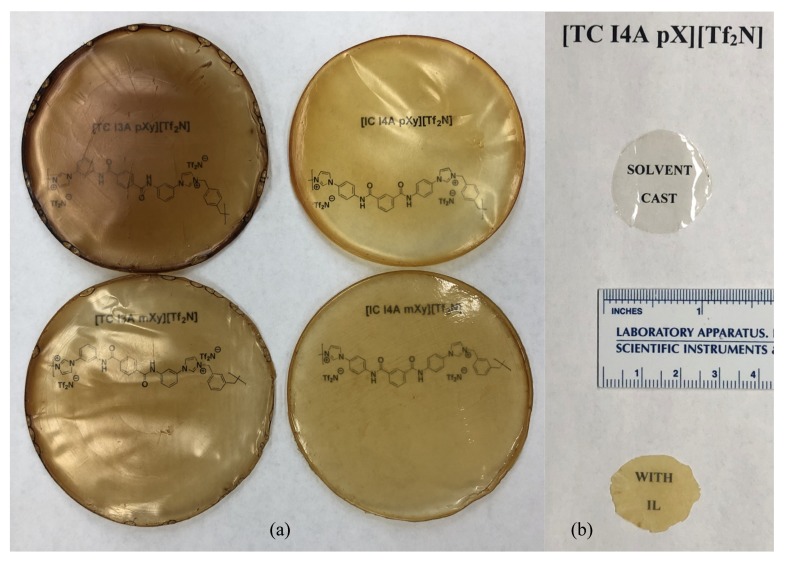
(**a**) Images of solvent cast films (d = 60 mm) of four PA-ionenes introduced in this work; (**b**) Comparison of [TC I4A pX][Tf_2_N] as a neat solvent cast film versus ionene-containing [Bnmim][Tf_2_N].

Solutions (5 wt%) of each PA-ionene, neat or with 1 eq. of [Bnmim][Tf_2_N] per repeat unit, were prepared using 0.4–0.6 g of neat polymer in DMAc. The solutions were prepared in 15 mL centrifuge tubes which were placed in a hot water bath for 2 h, to promote the dissolution and distribution of the solute. The tubes were then centrifuged for 5 min at 6000 rpm to separate any undissolved solids. Each solution was then poured into PTFE molds (d = 60 mm), which were then heated to 40 °C in a vacuum oven for 24 h to set the material. The temperature was slowly raised to 65 °C, 85 °C, and then 120 °C over the course of 72 h in order to remove remaining solvent. The films were then peeled from the mold to yield free-standing membranes. The resultant film thicknesses for these derivatives were 120–140 μm, as initially measured with digital calipers and later confirmed by SEM images of cross-sections (Figures S17–S19). It should be noted that derivative **1** was very mechanically durable and flexible when cast as much thinner films, contrary to other derivatives that required greater thicknesses for sufficient testing as unsupported membranes. Thus, [TC I4A pX][Tf_2_N] was solvent cast to as thin as 4.1 μm to demonstrate its mechanical stability and permeation consistency even as a thinner membrane. Casting of [IC I3A mX][Tf_2_N] and [TC I2A oX][Tf_2_N] derivatives (**2, 7**) was attempted but yielded brittle, paper-like materials which did not hold under pressure. This is likely due to poor entanglement or inability to properly align for H-bonding of consistent *meta*-connectivity for **2**, the strain of the *ortho*-connectivity along the backbone for **7**. Figure 4 shows the materials which were amenable to film formation, demonstrating flexibility and stability as thin, transparent membranes.

### 3.3. Gas Permeation Testing

Pure gas permeation measurements were performed to determine the gas separation performance of these aromatic PA-ionene membranes using a high-vacuum time lag measurement unit based on the constant-volume/variable-pressure method. Our previous work discusses the specifications and construction of the units [8,47]. All measurements were conducted at 20 °C and ~3 atm (~45 psia) feed pressure against initial downstream vacuum (<0.01 psia). Thin films were cast of derivatives **1**–**5** via the methods outlined above. The PA-ionenes with consistent connectivity along the backbone (i.e., [TC I4A pX][Tf_2_N] = *para-para-para*; [IC I3A mX][Tf_2_N] = *meta-meta-meta)* exhibited notably higher glass transitions and suitable mechanical stability in the *para*-case. The film of derivative **2** appeared opaque, paper-like, and thus brittle. Derivatives **3**–**6** also yielded transparent thin films with good properties, despite the inconsistent connectivity, but these did possess lower *T*_g_ values and broader distributions seen in the MALDI-TOF MS data. Thus, **1** was selected for comparison when IL is incorporated into the matrix. The addition of [Bnmim][Tf_2_N] improved the flexibility of the membranes, and altered the optical qualities of the films. This induced opacity may be attributed to the additional ordering and coordination which occurs when IL is added to the ionene. The IL was expected to regulate and increase the free volume, as indicated by an increase in *d*-spacing. However, a stark decrease in permeability was observed from the addition of IL. Therefore, derivatives **3**–**5** were tested as neat membranes. 

As shown in Table 3, PA-ionene **1** which contained consistent *para*-connectivity exhibited the best gas permeation performance as well as stability. This material had the highest *T*_g_ and was robust mechanically even as the thinnest film cast in this work (4.1 μm). To ensure consistency of permeation experiments, this permeation data was also collected with a membrane sample with a thickness in the same range as **3**–**6**. The CO_2_ permeability was 11 barrer, which yielded high selectivity relative to N_2_ and CH_4_. The stoichiometric incorporation of IL into the matrix drastically reduced permeability for all gases, particularly for CO_2_ which decreased by nearly 95%. Derivative **1 + IL** exhibited very low gas permeability, which may be due to increased interactions between chains tightening the matrix. While other similar PI-ionenes and PA-ionenes have shown improved performance with the addition of IL, these PA-ionenes form a denser polymer matrix when neat due to their primarily aromatic character and combined H-bonding, ionic, and stacking intermolecular interactions. Rather than pushing chains apart, in this case, the introduction of IL may improve coordination of the ionene and tighten the interchain spacing or fill free volume, and inhibiting gas permeation. Derivatives **3** and **4** exhibited comparable *d*-spacing yet **4** was notably denser, which is reflected by greater gas permeability in **3** versus **4**. As both have *para*-connectivity from the terephthalamide segment, **3** has adjacent *meta*-connectivity in the imidazole-amide connection with the *meta*-xylyl linkage, while **4** contains adjacent *meta*-connectivity in the imidazole-amide connection with the *para*-xylyl linkage. This suggests that further deviation from consistent *para*-connectivity corresponds to decreasing permeability and that connectivity within these various repeat unit segments does influence the efficiency of chain packing. Comparing the three TC-based ionenes (**1**, **3**, **4**), the initial [TC I4A pX][Tf_2_N] ionene exhibited CO_2_ permeability which was nearly 3× or 4.5× higher than **3** and **4**, respectively. However, these all showed high selectivity for CO_2_, as N_2_ and CH_4_ permeability was not promoted by this system. The spacing between the amide groups via *para*-connectivity may improve H-bonding and chain interactions, demonstrated by the greater mechanical stability of these materials. Shifting to derivative **5**, which contains the isophthalamide linkage adjacent to *para*-connectivity between the imidazolium and amide groups, obtained relatively low permeabilities for all the gases with superior CO_2_ selectivities over N_2_ and CH_4_ gases, plausibly due to the high density despite the increased interchain *d*-spacing value compared to **3** and **4**.

## 4. Conclusions

A series of seven bis-imidazole amide monomers that were polymerized with xylyl linkages to form imidazolium PA-ionene isomers with varied connectivity. The synthetic methods were simple and each product was obtained in high yields. The influence of connectivity in each segment was probed through thermal and structural characterization, and four of the seven polymers provided suitable very thin, unsupported membranes. [TC I4A pX][Tf_2_N] with consistent *para*-connectivity corresponded to the most stable, robust material with the highest CO_2_ permeability and also yielded superior CO_2_/CH_4_ and CO_2_/N_2_ selectivities. Nonetheless, the addition of free IL significantly reduces the permeability of [TC I4A pX][Tf_2_N], plausibly because of dense amorphous chain packing regardless to the free IL content, which was in good agreement with the density measurements. Derivatives **3**–**5**, which incorporated varied connectivity along the PA-ionene backbone, exhibited moderate CO_2_ permeabilities without significant loss in selectivities. While certain isophthalamide-based systems were less mechanically stable and permeable, derivative **5**, which contains the isophthalamide linkage adjacent to *para*-connectivity, displayed enhanced CO_2_/N_2_ and CO_2_/CH_4_ selectivities. This work highlights the beneficial features of different amide linkages in the ionene backbone. Therefore, future investigations will explore more on the incorporation of other functional groups and spacing of the alternating ionic and functional groups.

## Figures and Tables

**Table 1:** Reactant Amounts and Polymer Yields for all seven PA-ionenes.

ID	PA-Ionene	Bis-Imidazole	Amount	Linkage	Amount	LiTf_2_N	Product Yield	Yield
**1**	[TC I4A pX][Tf_2_N]	TC I4A	10.1 g | 22.6 mmol	pDCX	3.95 g | 22.6 mmol	16.2 g	21.5 g	86%
**2**	[IC I3A mX][Tf_2_N]	IC I3A	8.00 g | 17.8 mmol	mDCX	2.50 g | 5.57 mmol	12.8 g	14.1 g	71%
**3**	[TC I3A mX][Tf_2_N]	TC I3A	2.50 g | 5.57 mmol	mDCX	0.98 g | 5.57 mmol	4.00 g	3.89g	62%
**4**	[TC I3A pX][Tf_2_N]	TC I3A	2.50 g | 5.57 mmol	pDCX	0.98 g | 5.57 mmol	4.00 g	4.31 g	70%
**5**	[IC I4A pX][Tf_2_N]	IC I4A	2.00 g | 4.46 mmol	pDCX	0.78 g | 4.46 mmol	3.20 g	2.76 g	56%
**6**	[IC I4A mX][Tf_2_N]	IC I4A	2.00 g | 4.46 mmol	mDCX	0.78 g | 4.46 mmol	3.20 g	2.41 g	49%
**7**	[TC I2A oX][Tf_2_N]	TC I2A	1.50 g | 3.35 mmol	oDCX	0.58 g | 3.35 mmol	2.40 g	2.84 g	76%

**Table 2:** Summary of thermal, structural, and molecular weight data for PA-ionenes.

ID	PA-Ionene	IL (Equivalents)	*T*_g_ (°C)	*d*-Spacing (Å)	Density (g/cm^3^)	M_N_ (kDa)
**1**	[TC I4A pX][Tf_2_N]	Neat	136.1	4.07	1.517	60.4
**1 + IL**		[Bnmim][Tf_2_N] (1)	-	4.26	1.575	*
**2**	[IC I3A mX][Tf_2_N]	Neat	133.5	4.35	1.443	62.5
**2 + IL**		[Bnmim][Tf_2_N] (1)	-	4.55	1.259	*
**3**	[TC I3A mX][Tf_2_N]	Neat	54.5	4.36	1.388	59.9
**4**	[TC I3A pX][Tf_2_N]	Neat	74.7	4.39	1.427	75.4
**5**	[IC I4A pX][Tf_2_N]	Neat	112.0	4.97	1.505	66.4
**6**	[IC I4A mX][Tf_2_N]	Neat	115.7	5.09	1.462	76.1
**7**	[TC I2A oX][Tf_2_N]	Neat	76.4	4.47	1.577	117.7

* MALDI-TOF MS analysis was not performed for derivatives containing IL, as the same polymer from **1** and **2** was used in the preparation of samples **1 + IL** and **2 + IL** and thus has the same M_N_ value.

**Table 3:** Pure gas permeability and permselectivity data for PA-ionenes at 20 °C and ~3 atm.

ID	PA-Ionene		Permeability and Selectivity Data ^a,b^				
			*P*_CO2_	*P*_N2_	*P*_CH4_	*α*_CO2/N2_	*α*_CO2/CH4_
**1**	[TC I4A pX][Tf_2_N]	Neat	11.00 ± 0.02	0.34 ± 0.02	0.23 ± 0.01	32.6 ± 1.9	46.9 ± 2.0
**1 + IL**		[Bnmim][Tf_2_N]	0.403 ± 0.06	0.112 ± 0.002	0.087 ± 0.031	3.6 ± 0.5	4.6 ± 1.8
**3**	[TC I3A mX][Tf_2_N]	Neat	3.91 ± 0.04	0.096 ± 0.004	0.122 ± 0.003	40.9 ± 1.8	30.5 ± 0.08
**4**	[TC I3A pX][Tf_2_N]	Neat	2.46 ± 0.03	0.082 ± 0.005	0.103 ± 0.003	30.2 ± 1.4	23.9 ± 0.08
**5**	[IC I4A pX][Tf_2_N]	Neat	1.69 ± 0.05	0.046 ± 0.004	0.039 ± 0.005	36.6 ± 3.4	43.3 ± 5.7

^a^ Permeability reported in barrer, where 1 barrer = 10^−10^ [cm^3^ (STP) cm]/(cm^2^ s cm Hg). ^b^ Error analysis for permeability was calculated from variation between multiple gas permeation runs. The propagation of this error was calculated for the selectivity ratios.

## Supplementary Materials

The following are available online at https://www.mdpi.com/2077-0375/10/3/51/s1. Figure S1: ^1^H-NMR spectrum for TC I4A monomer, Figure S2: ^1^H-NMR spectrum for IC I3A monomer, Figure S3: ^1^H-NMR spectrum for TC I3A monomer, Figure S4: ^1^H-NMR spectrum for IC I4A monomer, Figure S5: ^1^H-NMR spectrum for TC I2A monomer, Figure S6: ^1^H-NMR spectrum for [TC I4A pX][Tf2N], Figure S7: ^1^H-NMR spectrum for [IC I3A mX][Tf2N], Figure S8: ^1^H-NMR spectrum for [TC I3A pX][Tf2N], Figure S9: ^1^H-NMR spectrum for [TC I3A mX][Tf2N], Figure S10: ^1^H-NMR spectrum for [IC I4A mX][Tf2N], Figure S11: ^1^H-NMR spectrum for [IC I4A pX][Tf2N], Figure S12: ^1^H-NMR spectrum for [TC I2A oX][Tf2N], Figure S13: MALDI-TOF spectra for all neat polyamide-ionenes, Figure S14: DSC cycles for all neat derivatives, Figure S15: Section of DSC cycles where Tg values are observed, Figure S16: TGA data for a selection of neat polyamide-ionenes, Figure S17: Cross-section of freeze-fractured, solvent-cast membrane of [TC I4A pX][Tf2N] at 9500X magnification. Thickness was measured to be 4.17 microns, Figure S18: Cross-section of freeze-fractured, solvent-cast membrane of [IC I3A mX][Tf2N] at 300X magnification. Thickness was measured to be 68.7 microns, Figure S19: Cross-section of freeze-fractured, solvent-cast membrane of [IC I3A mX][Tf2N] at 150X magnification. Thickness was measured to be 68.7 microns.
