# Supplementary material for: Synthesis and Performance of Aromatic Polyamide Ionenes as Gas Separation Membranes
Source: Membranes (Basel). 2020 Mar 22;10(3):51. doi: 10.3390/membranes10030051 (PMC7143725; doi:10.3390/membranes10030051)
Supplement: Supplementary file 1 [file membranes-10-00051-s001.zip › membranes-744948-SI.docx]

Supplementary Materials: Synthesis and Performance of Aromatic Polyamide Ionenes as Gas Separation Membranes

Kathryn E. O’Harra ^1^, Irshad Kammakakam ^1^, Danielle M. Noll ^1^, Erika M. Turflinger ^1^, Grayson P. Dennis ^1^, Enrique M. Jackson ^2^ and Jason E. Bara ^1,^*

^1^ Department of Chemical & Biological Engineering, University of Alabama, Tuscaloosa, AL 35487-0203 USA; keoharra@crimson.ua.edu (K.E.O.); ikammakakam@eng.ua.edu (I.K.); dmnoll@crimson.ua.edu (D.M.N.); emturflinger@crimson.ua.edu (E.M.T.); gpdennis@crimson.ua.edu (G.P.D.); [jbara@eng.ua.edu](mailto:jbara@eng.ua.edu) (J.E.B.)

^2^ NASA Marshall Space Flight Center, Huntsville, AL 35812, USA; [enrique.m.jackson@nasa.gov](mailto:enrique.m.jackson@nasa.gov) (E.M.J.)

***** Correspondence: jbara@eng.ua.edu

Received: 27 February 2020; Accepted: 20 March 2020; Published: date

Included are relevant supporting data referenced in the manuscript: “Synthesis and Performance of Aromatic Polyamide Ionenes as Gas Separation Membranes”. NMR, MALDI-TOF MS, DSC, TGA, XRD, and SEM results are shown in the following sections.


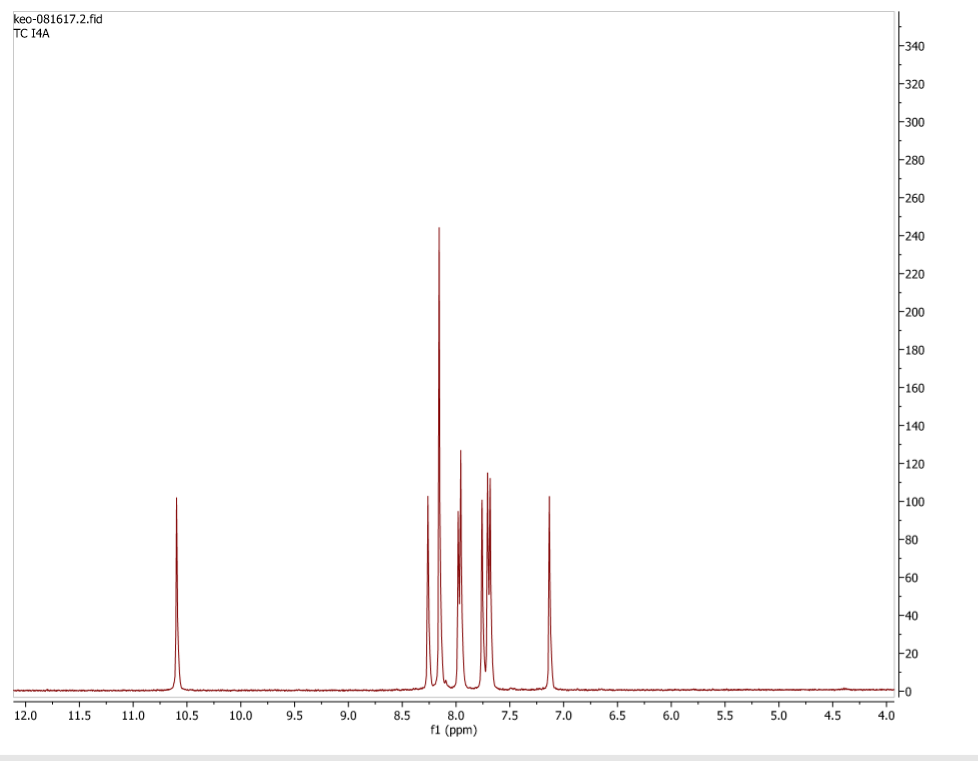
 The ^1^H-NMR spectra for the monomers and polymers outlined in the manuscript are included below, with corresponding structures included. Assignments and specific *ppm* assignments and splitting patterns are outlined in the manuscript.

**Figure S1:** ^1^H-NMR spectrum for TC I4A monomer.


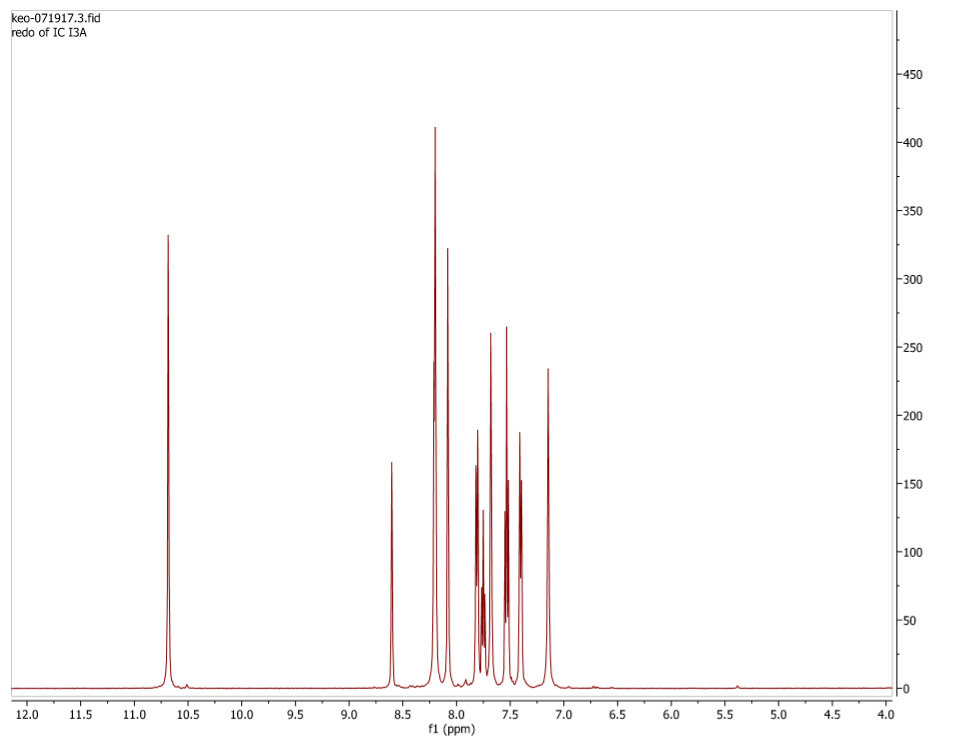


**Figure S2:** ^1^H-NMR spectrum for IC I3A monomer.


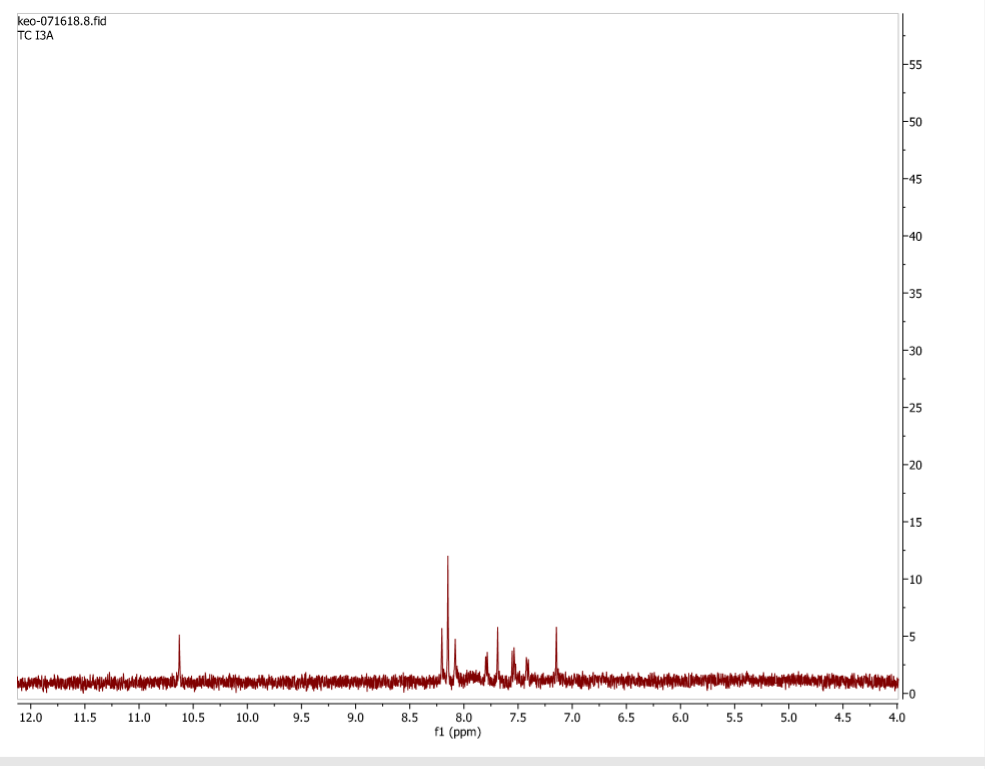
**Figure S3:** ^1^H-NMR spectrum for TC I3A monomer.

**Figure S4:** ^1^H-NMR spectrum for IC I4A monomer.
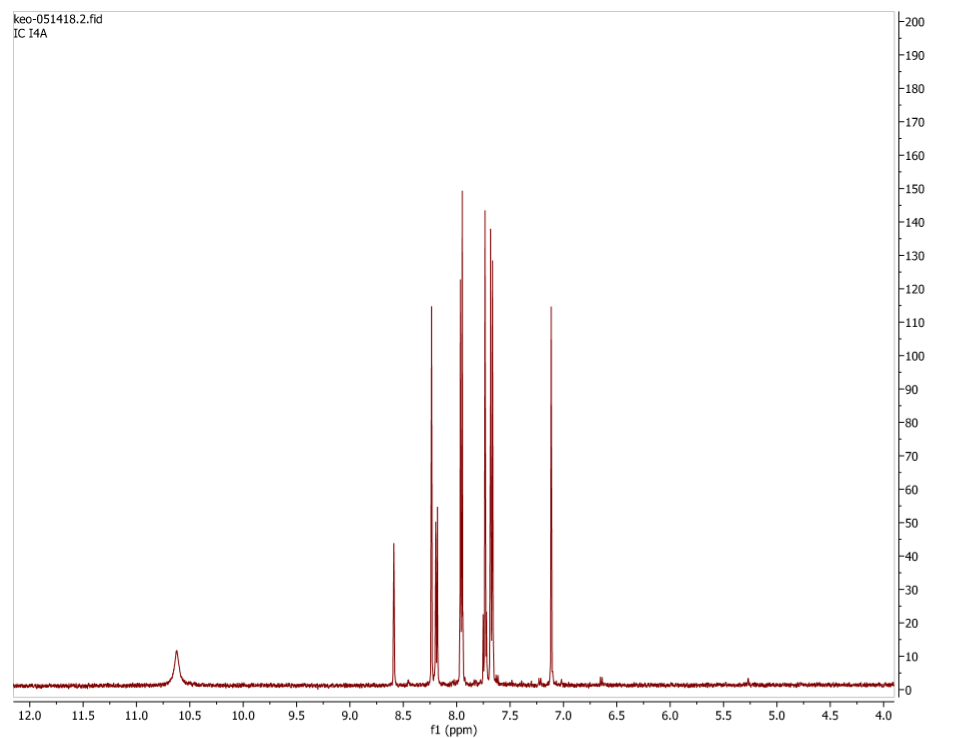


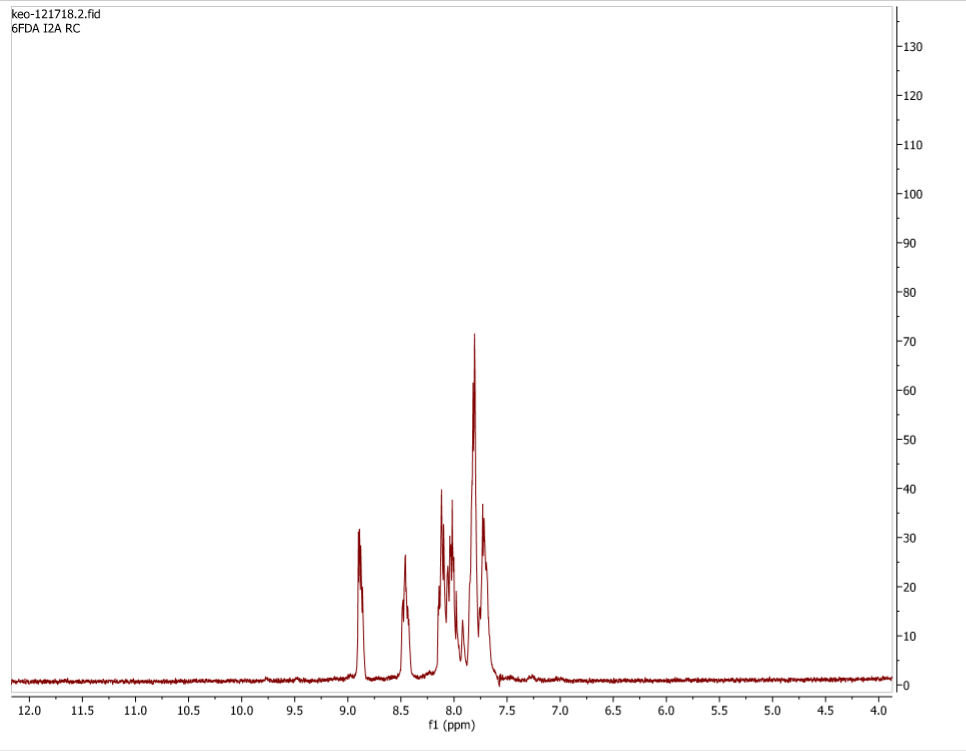
**Figure S5:** ^1^H-NMR spectrum for TC I2A monomer.


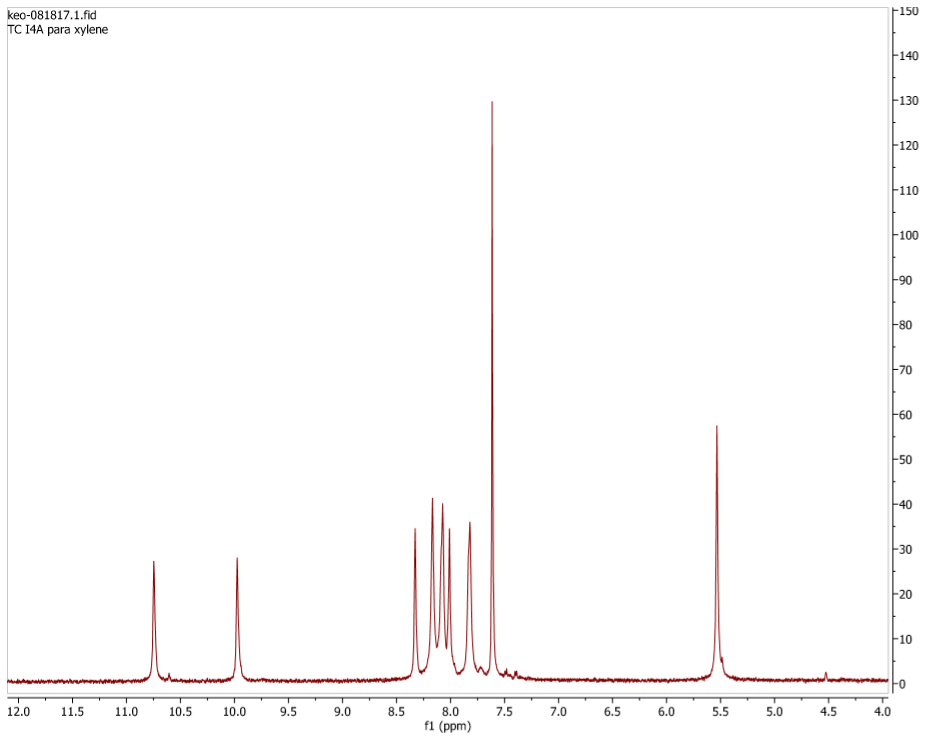


**Figure S6: ^1^H-NMR spectrum for [TC I4A pX][Tf_2_N].**


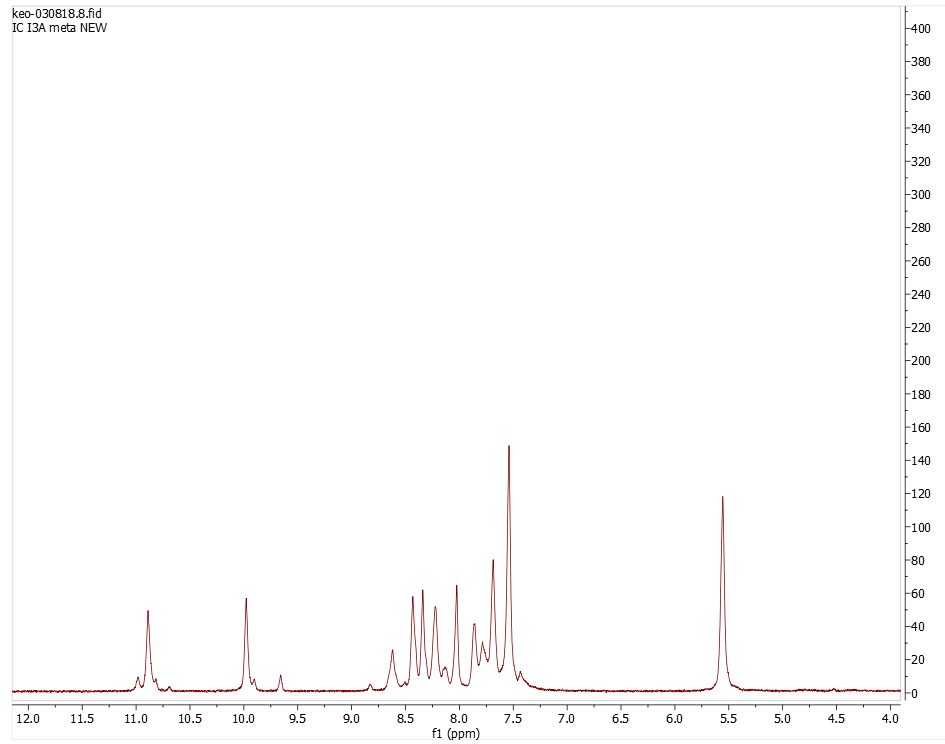


**Figure S7:** ^1^H-NMR spectrum for [IC I3A mX][Tf_2_N].


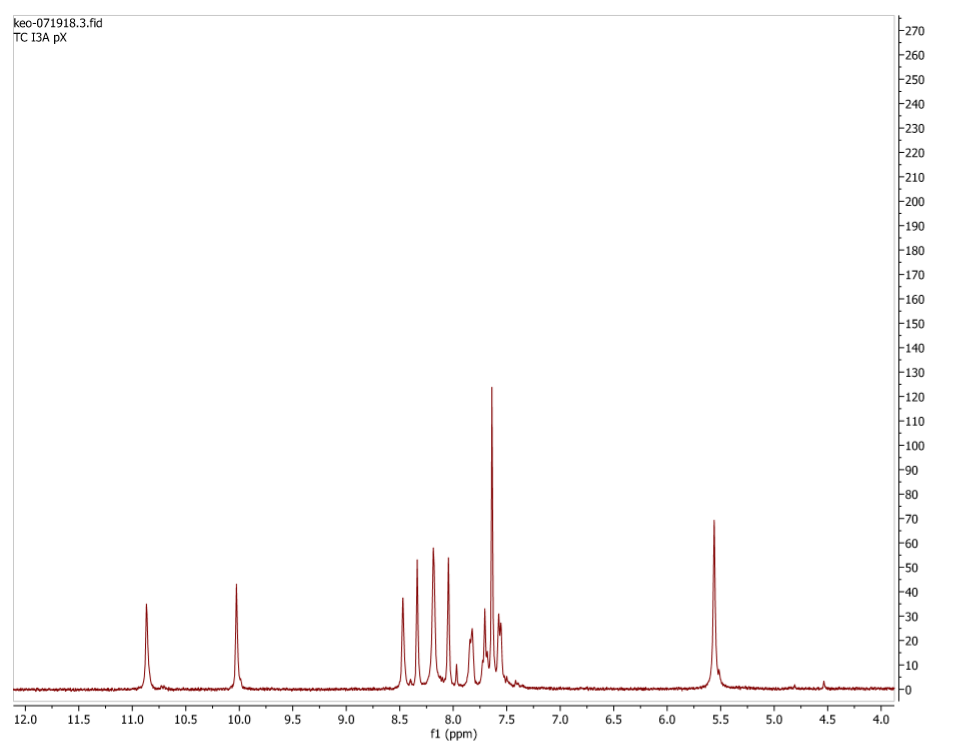


**Fig ure S8:** ^1^H-NMR spectrum for [TC I3A pX][Tf_2_N].


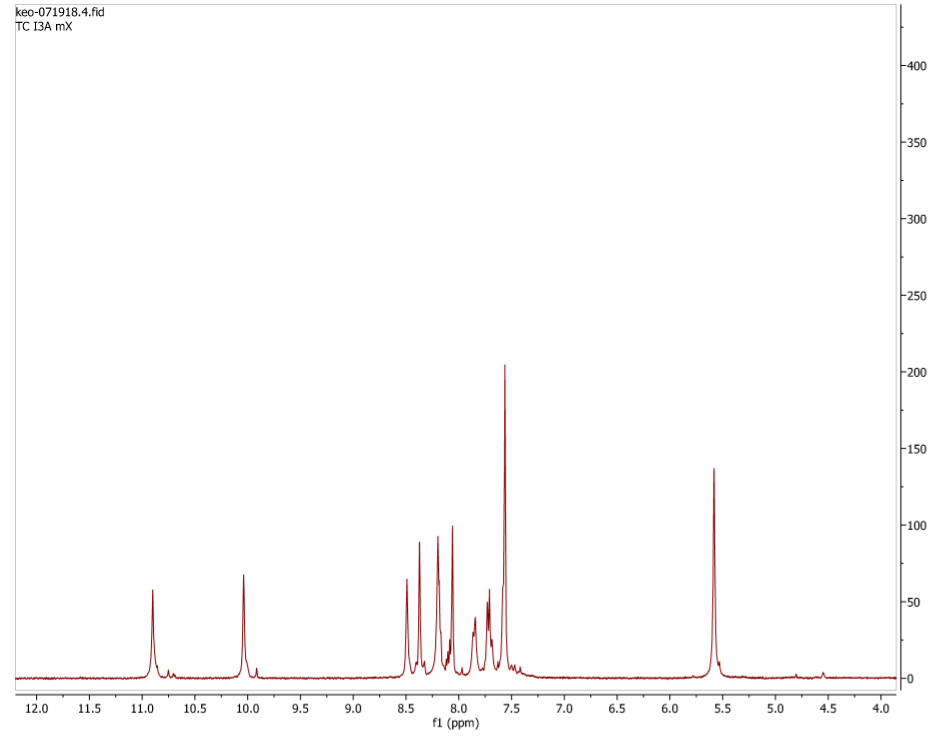


**Figure S9:** ^1^H-NMR spectrum for [TC I3A mX][Tf_2_N].


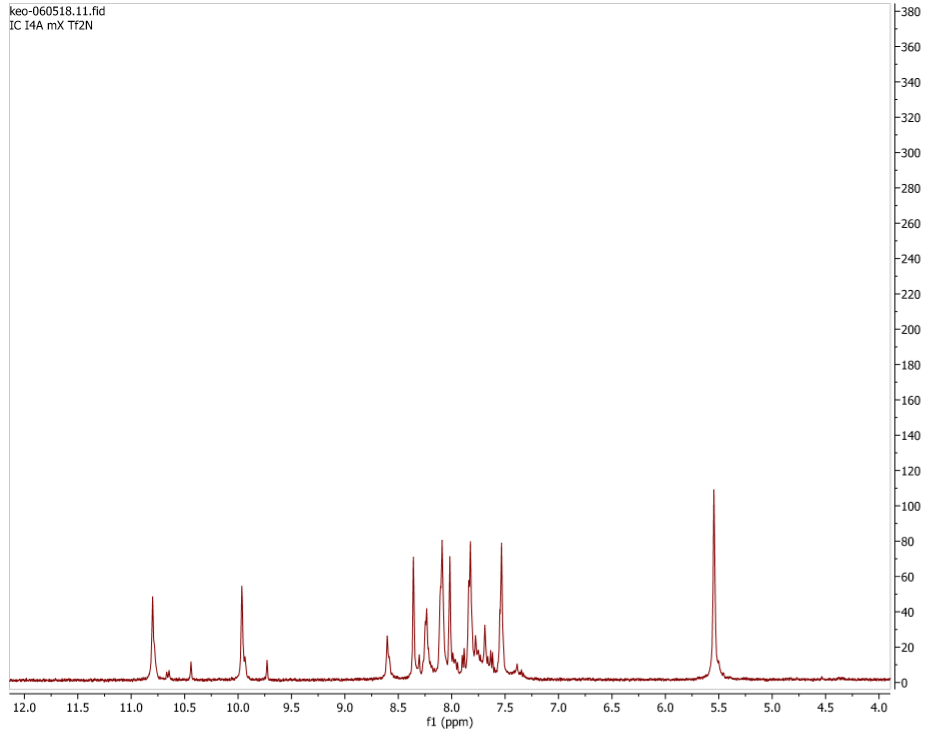


**Figure S10: ^1^H-NMR spectrum for [IC I4A mX][Tf_2_N].**


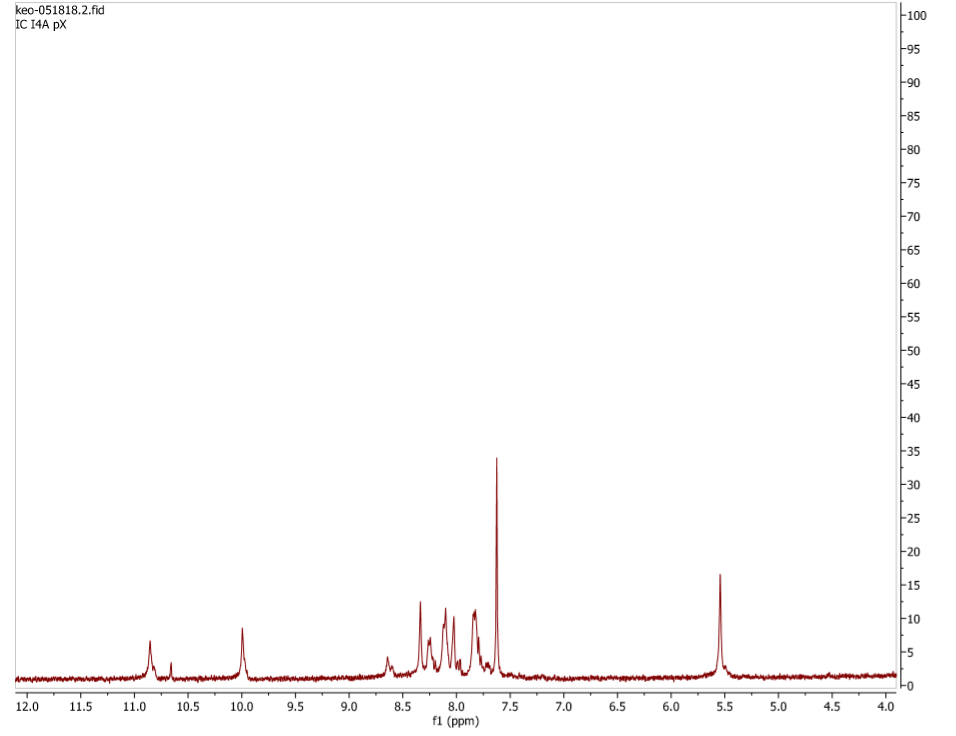


**Figure S11: ^1^H-NMR spectrum for [IC I4A pX][Tf_2_N].**


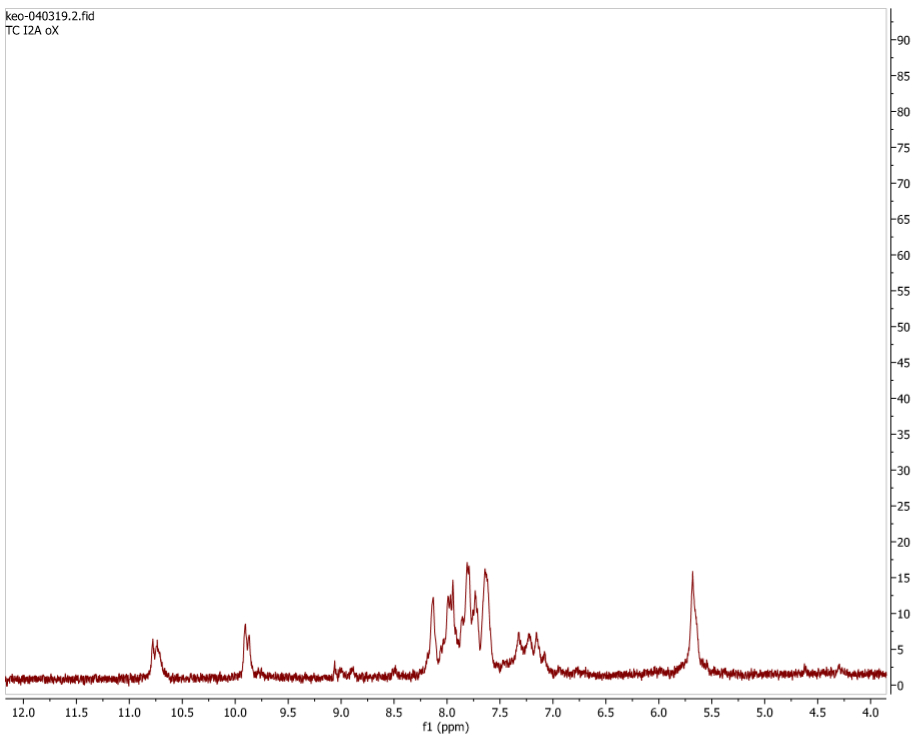


**Figure S12: ^1^H-NMR spectrum for [TC I2A oX][Tf_2_N].**

**Mass Spectroscopy**

The number average molecular weight (M_N_) was determined via MALDI-TOF mass spectrometry. Each neat polyamide ionene was dissolved in DMAc and deposited on the plate, with BSA protein in MeOH as the standard for instrument calibration. THAP or DCTB (in MeOH) matrices were used to promote ionization.

**Figure S13: MALDI-TOF spectra for all neat polyamide-ionenes.**

**Differential Scanning Calorimetry (DSC) and Thermogravimetric Analysis (TGA)**

The glass transition temperatures (T_g_) were determined by DSC, as discussed in the manuscript. The temperature was increased from 30 – 300 °C, and all thermographs are plotted “exo up”. In the first run, the scan was cycled from 30 °C to 300 °C, then back down to 30 °C. Figure S14 shows the full cycle, whereas Figure S15 highlights the T_g_.

|  |  |
| --- | --- |

**Figure S14:** DSC cycles for all neat derivatives.

| **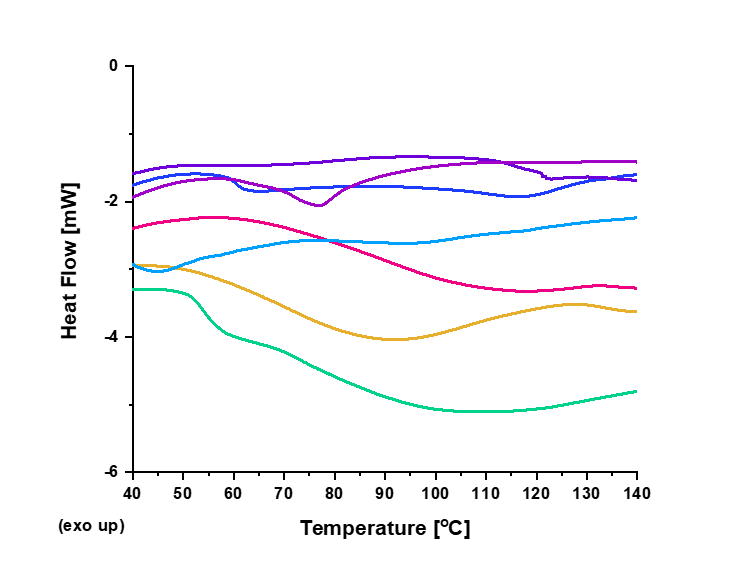** |  |
| --- | --- |

**Figure S15:** Section of DSC cycles where Tg values are observed.

**Figure S16:** TGA data for a selection of neat polyamide-ionenes.

**Scanning Electron Microscopy (SEM) Images**

Images were captured on a Thermo Scientific Apreo FE-SEM. Membranes (derivatives 1-2) were solvent cast, then fractured in liquid N_2_. The cross-section was captured at magnifications between 150X-9500X.


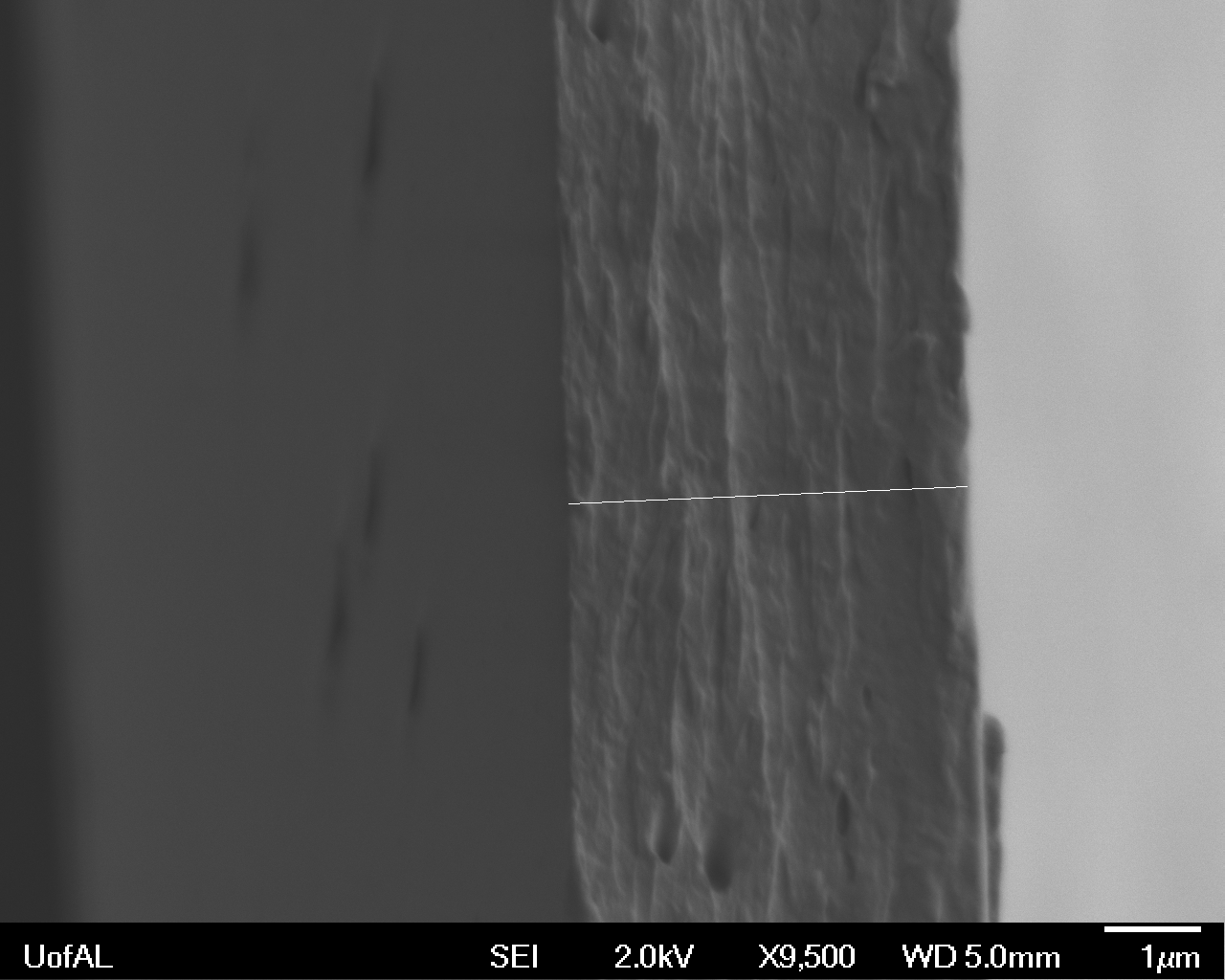


**Figure S17:** Cross-section of freeze-fractured, solvent-cast membrane of [TC I4A pX][Tf_2_N] at 9500X magnification. Thickness was measured to be 4.17 microns.


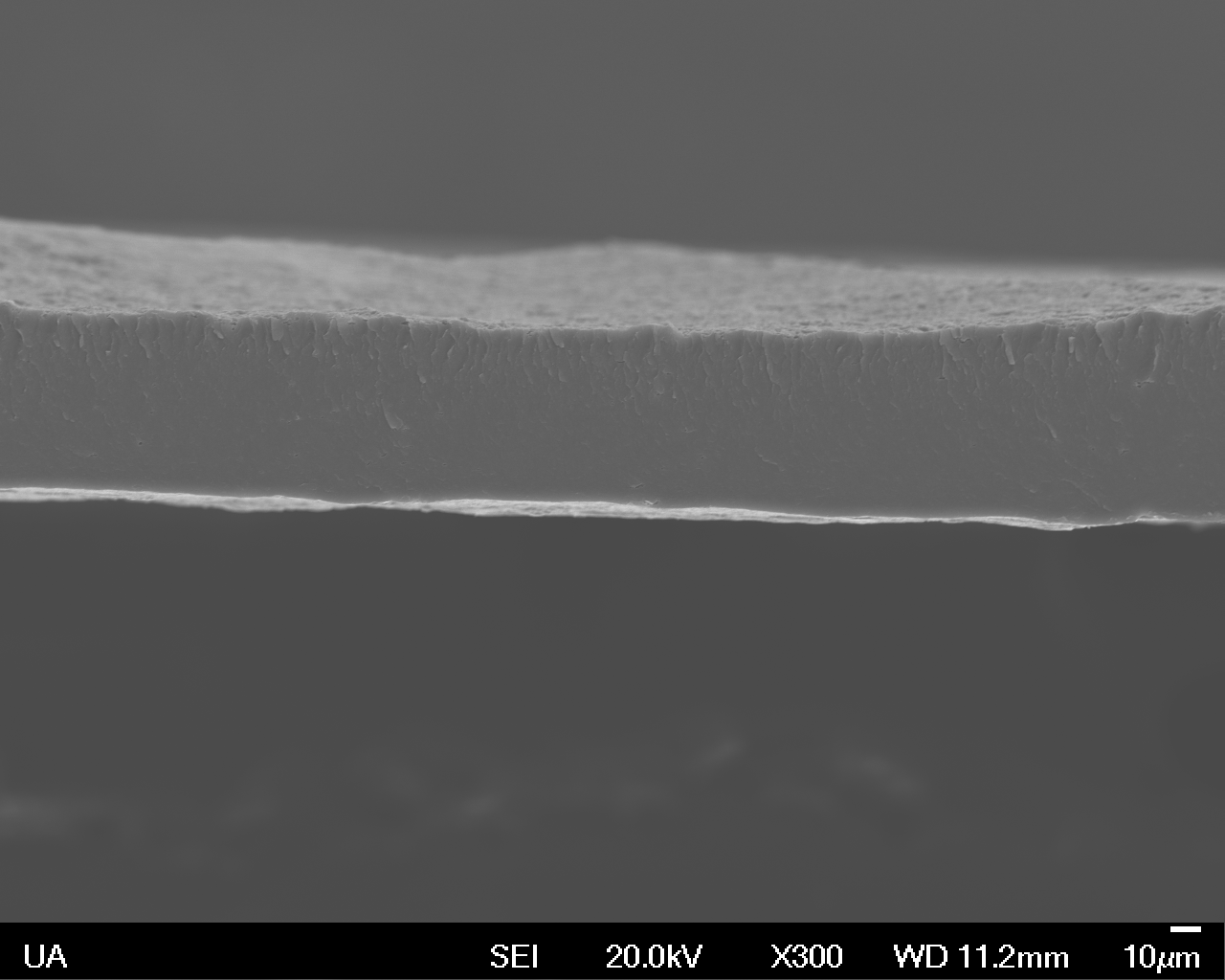


**Figure S18:** Cross-section of freeze-fractured, solvent-cast membrane of [IC I3A mX][Tf_2_N] at 300X magnification. Thickness was measured to be 68.7 microns.


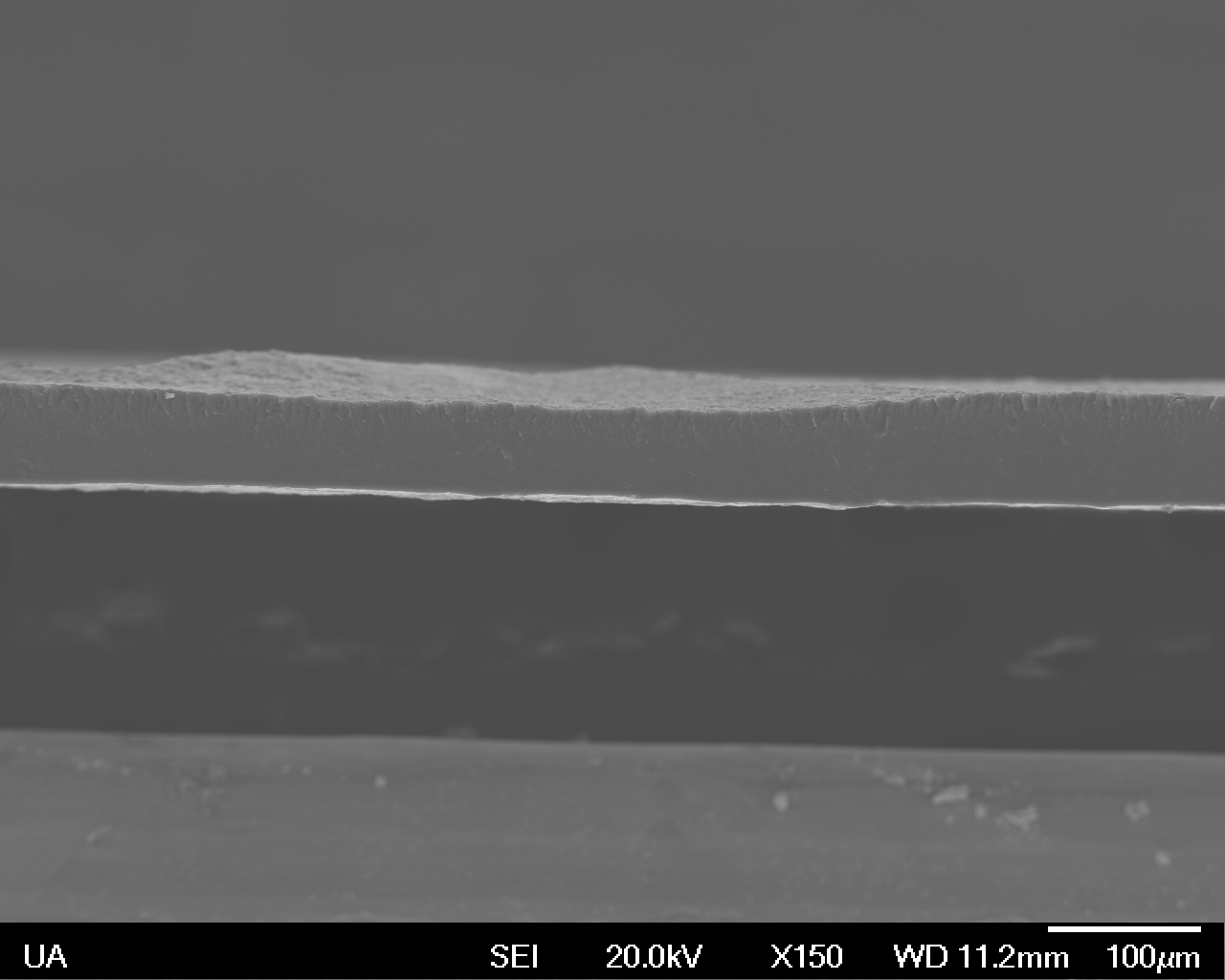


**Figure S19:** Cross-section of freeze-fractured, solvent-cast membrane of [IC I3A mX][Tf_2_N] at 150X magnification. Thickness was measured to be 68.7 microns.

| 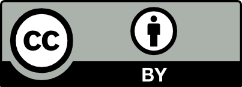 | © 2020 by the authors. Submitted for possible open access publication under the terms and conditions of the Creative Commons Attribution (CC BY) license (http://creativecommons.org/licenses/by/4.0/). |
| --- | --- |
